# From clones to synthetic embryos

**DOI:** 10.1093/lifemedi/lnad038

**Published:** 2023-10-24

**Authors:** Carlos A Pinzón-Arteaga, Leqian Yu

**Affiliations:** Department of Molecular Biology, University of Texas Southwestern Medical Center, Dallas, TX 75390, United States; State Key Laboratory of Stem Cell and Reproductive Biology, Institute of Zoology, Chinese Academy of Sciences, Beijing 100101, China; Institute for Stem Cell and Regeneration, Chinese Academy of Sciences, Beijing 100101, China; Beijing Institute for Stem Cell and Regenerative Medicine, Beijing 100101, China

## Cloning V1.0 to V2.0

**Figure 1.**
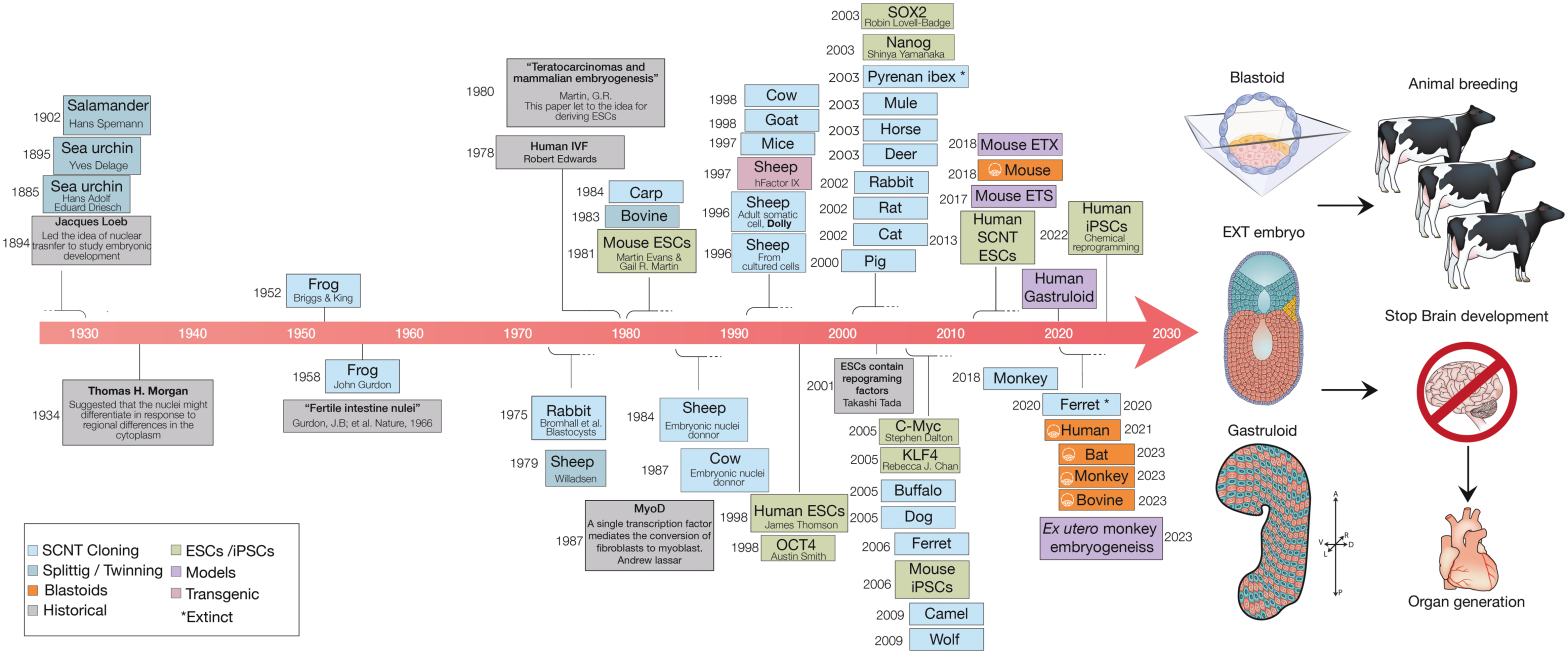
History timeline and future applications.

The idea of using nuclear transfer as a method to study embryonic development can be traced back to the beginnings of experimental embryology in the late 1890s, by 1952 Roberts Briggs and Thomas King showed that normal hatched tadpoles could be obtained by transplanting the nucleus of a blastula cell to the enucleated frog eggs. Following Briggs and King’s work, in 1962, John Gurdon showed that even the nuclei of the most differentiated cells could support normal development. This seminal work inspired the work of many others throughout the 1970s up to the birth of Dolly, the first demonstration of a live mammalian offspring cloned from the nucleus of an adult somatic cell (SCNT), by Ian Wilmut and Keith Campbell at the Roslin Institute in 1996. SCNT cloning has since then been adapted to several species (Fig. 1).

At the same time, another revolution was taking place, during the 1970s Martin Evans and Gail R. Martin thanks to their work with teratocarcinoma and embryonal carcinoma cells (EC), both groups were the first to derive mouse embryonic stem cells (ESCs) from mouse embryos in 1981. Leading years later to the first human ESC line derivation by James Thomson in 1998, the advent of mouse ESCs inspired several groups during the 1990s to identify the factors and signaling mechanisms that were essential for the pluripotency of the mammalian embryo. One of these groups was Shinya Yamanaka, after some years of work in ESCs self-renew factors such as Nanog, he used the convergence of three paths of science to derive the first induced pluripotent stem cells (iPSCs).

The availability of ESCs and trophoblast stem cells (TSCs) derived by Janeth Rossant, leading years latter to the development of the first “synthetic” mouse embryos (ETS and ETX embryo) by Magdalena Zernicka-Goetz [1] and then followed a year later by Nicolas Rivron with the first assembled mouse blastocyst like structures termed blastoids [2]. Now blastoids from several species have been reported, including human [3], monkey [4], and cow [5] (Fig. 1).

SCNT uses the contents of the oocyte to reprogram a somatic cell into forming a blastocyst, while blastoids are made from reprogrammed iPSCs or pluripotent ESCs and are therefore considered to be “synthetic.” One key difference between both techniques is the ability to generate a theoretical infinite number of blastoids thanks to ESCs unlimited self-renew capacity, whereas SCNT is limited by the availability of oocytes and suffers from low developmental rates due to incomplete epigenetic reprogramming. This unique access to large numbers of blastocyst-like structures will have profound impact in our understanding of the mechanisms behind mammalian blastocysts formation and implantation. Eventually, as the fidelity of blastoids improves, it is likely that practical applications will appear, such as the ability to generate blastoid-derived animals “Cloning V2.0,” and because blastoids can be generated on a large scale, this potential biotechnology could significantly lower the costs of SCNT and increase the access to high-quality animal genetics around the world. Also, the knowledge these models will generate will likely improve the current *in vitro* fertilization (IVF) systems, generate new non-hormonal contraceptives, and improve pregnancy rates of IVF-produced embryos.

## Generate human organs?

In 2022, Jacob H. Hanna’s and Magdalena Zernicka-Goetz’s groups reported that synthetic mouse ETX embryos could undergo gastrulation, advance through key developmental milestones, and develop organ progenitors, including headfolds, a beating heart, somites, neural tube, and gut tube, within complex extraembryonic compartments resembling a day 8.5 post-fertilization mouse natural embryo. This achievement raises the possibility of another application for synthetic embryos: generating human organs for regenerative medicine.

Currently, neither the use of pluripotent stem cells for *in vitro* directed differentiation nor organoids can fully recreate the complex environment of *in vivo* development with multicellular interactions. This poses a challenge in obtaining integrated organs with functionality *in vitro*. However, if functional synthetic embryos containing all lineages of early embryos can develop *in vitro* and reproduce the *in vivo* developmental environment, it may be possible to obtain functional organs or precursor cells for transplantation therapies.

However, the translation of this work to humans won’t be easy. The key question is whether these synthetic embryos should be considered actual embryos, and if answered affirmatively, critical ethical issues will arise. In medicine, brain death is considered a sign of death. On the other hand, it has been observed that headless embryos can still develop normally without impacting the development of other organs. This raises the question of whether it is possible to selectively block neuron development in human synthetic embryos while allowing the development of other organs. If this can be achieved, it could potentially pave the way for ethically producing transplantable functional organs from a patient’s own iPSCs through synthetic embryos.

In conclusion, although synthetic embryos offer fascinating applications, they are still in a very preliminary stage, and many technical challenges remain to be overcome. One major hurdle is the extremely low developmental capacity of synthetic embryos compared to natural embryos, which can be attributed to various factors. However, with a growing understanding of early human development, advancements in stem cell- and embryo-culture techniques, and the development of synthetic embryo generation methods, we believe that this goal will be achieved in the near future.
